# Association between cannabis use and ocular inflammatory disease: a large-scale cohort study

**DOI:** 10.1186/s12348-025-00544-z

**Published:** 2025-11-11

**Authors:** Natan Lishinsky, Ortal Buhbut, Shoham Kubovsky, Nir Erdinest, Radgonde Amer, Zvi Gur

**Affiliations:** 1 Faculty of Medicine, Hebrew University of Jerusalem, Jerusalem, Israel; 2 Department of Ophthalmology, Soroka Medical Center, Ben Gurion University of the Negev, Beer-Sheva, Israel; 3 Department of Ophthalmology, Hadassah Medical Organization and Faculty of Medicine, Hebrew University of Jerusalem, Jerusalem, Israel; 4 Hadassah Medical Center, Jerusalem, Israel

**Keywords:** Cannabis, Marijuana, Uveitis, Iridocyclitis, Ocular inflammation, Retinal vasculitis, Panuveitis, Real-world evidence, TriNetX

## Abstract

**Objective:**

Cannabis use has increased substantially worldwide, yet its association with inflammatory eye diseases remains poorly understood. This study evaluated whether cannabis users have higher risk of developing uveitis and related inflammatory ocular conditions compared to non-users.

**Methods:**

We conducted a retrospective cohort study using the TriNetX Global Collaborative Network database. Adult patients with documented cannabis-related disorders were propensity score-matched 1:1 to patients with no cannabis use, excluding individuals with prior diagnoses that could independently cause uveitis. The primary outcome was the incidence of any uveitis. Secondary outcomes included specific uveitis subtypes, retinal vasculitis, and choroidal degeneration, assessed starting 1 year after cohort entry. Kaplan-Meier survival analysis and Cox proportional hazards models compared outcomes between groups.

**Results:**

After propensity matching, 1,156,655 cannabis users were compared with 1,156,655 matched non-users. Cannabis use was associated with significantly increased risk of any uveitis (hazard ratio [HR] 1.8, 95% confidence interval [CI] 1.65–1.95, *p* < 0.0001). Specific uveitic conditions showed higher relative risks: panuveitis demonstrated the strongest association (HR 3.64, 95% CI 2.24–5.91, *p* < 0.0001), followed by choroidal degeneration (HR 3.29, 95% CI 1.74–6.23, *p* < 0.0001) and retinal vasculitis (HR 3.27, 95% CI 1.81–5.89, *p* < 0.0001).

**Conclusions:**

Cannabis use was associated with statistically and clinically significant increased risk of ocular inflammatory diseases, particularly those affecting the posterior eye segment. These findings have important implications for ophthalmologic screening and patient counseling as cannabis use becomes more widespread.

## Introduction

Uveitis is a heterogeneous group of intraocular inflammatory disorders affecting the uveal tract and adjacent structures, representing a significant cause of preventable blindness globally. Clinical presentation varies by anatomical classification: anterior uveitis typically presents with pain, redness, and photophobia; intermediate uveitis with floaters and mild vision loss; while posterior uveitis and panuveitis cause more severe visual impairment [1–4]. 

Etiologies include idiopathic, autoimmune, and infectious causes [2, 3] Systemic associations including HLA-B27 spondyloarthropathies, sarcoidosis, and Behçet’s disease are frequent in noninfectious uveitis [1, 5, 6]. Management varies by etiology and location, ranging from topical corticosteroids for anterior uveitis to systemic immunosuppressive agents and biologics like adalimumab for posterior and panuveitis [1, 7]. 

Despite increasing cannabis usage rates-with 18% of Americans reporting use in 2019-the relationship between cannabis and uveitis has not been systematically investigated in large-scale epidemiological studies [8, 9]. While the endocannabinoid system plays a role in immune regulation and ocular inflammation, available data are primarily preclinical. Experimental studies demonstrate that Cannabinoid receptor 2.

receptor activation can reduce leukocyte-endothelial adhesion and decrease pro-inflammatory mediators in endotoxin-induced uveitis models, but these findings have not been validated in human populations [10, 11]. 

Using the TriNetX Global Collaborative Network, this study investigates the incidence of uveitis and related inflammatory ocular conditions in cannabis users compared with matched control patients in the outpatient setting.

## Methods

### Study design and data source

This retrospective cohort study was conducted using the TriNetX Global Collaborative Network, a federated research platform that, as of June 2025, compiles de-identified electronic health records from more than 150 healthcare organizations worldwide. The platform offers access to a wide range of clinical data, including patient demographics, diagnoses, procedures, medications, laboratory results, and healthcare utilization. All data is de-identified in compliance with the Health Insurance Portability and Accountability Act (HIPAA). Diagnoses were coded using the International Classification of Diseases, 10th Revision, Clinical Modification (ICD-10-CM), while procedures were identified using Current Procedural Terminology (CPT) codes. Medications were classified using RxNorm.

### Cohort definition

We constructed two cohorts of adults (age ≥ 18 years). The Cannabis Use cohort included patients with any documentation of cannabis-related disorders or use. This was defined by an ICD-10-CM diagnosis code for cannabis-related disorders (F12), relevant procedure codes for cannabinoid use or testing (e.g., CPT 80349 for natural cannabinoids), or entries for cannabinoid medications (RxNorm code 1976). The Control cohort included patients who had received ophthalmic care (such as an eye examination or procedure, identified by CPT code 1012793 for ophthalmology services or ICD-10-CM Z01.0 for eye exams and Z13.5 for eye screening) but had no documented cannabis use in their records. To focus on new onset ocular inflammation, we excluded from both cohorts any patients with a history of uveitis or ocular inflammatory disease prior to the index event.

Each patient’s index event was defined as the first qualifying encounter: for the cannabis cohort, the first recorded diagnosis/procedure indicating cannabis use; for controls, an encounter for an eye examination or related ophthalmic visit (with no prior cannabis use). We imposed a 1-year “washout” period after the index date during which outcomes were not counted, to ensure we were capturing incident (new) cases of eye inflammation rather than pre-existing conditions.

### Propensity score matching

To reduce confounding, we employed propensity score matching (PSM) to balance baseline characteristics between the two cohorts. Using TriNetX’s built-in analytic tools, we performed 1:1 nearest-neighbor matching without replacement, using a caliper of 0.1 pooled standard deviations. The propensity model included demographics (age at index, sex, race/ethnicity) and key comorbidities known to influence inflammatory status or ocular health. These covariates included hypertensive disease (ICD-10-CM I10-I15), nicotine dependence (F17), diabetes mellitus (E08-E13), ischemic heart disease (I20-I25), and chronic kidney disease (N18) Covariate balance after matching was assessed using standardized mean differences (SMD), with an absolute SMD < 0.1 indicating good balance between groups.

### Outcomes

The primary outcome was the development of any inflammatory eye disease (uveitis or related conditions) during follow-up. This was defined as a composite of uveitis-related diagnoses including anterior uveitis/iridocyclitis (ICD-10-CM H20), intermediate/posterior uveitis such as chorioretinal inflammation (ICD-10-CM H30.9) and posterior cyclitis (ICD-10-CM H30.2), panuveitis (ICD-10-CM H44.11), retinal vasculitis (ICD-10-CM H35.06), and choroidal inflammation or degeneration (ICD-10-CM H31.1). Additional specific diagnoses of interest were sympathetic ophthalmia (ICD-10-CM H44.13), Vogt-Koyanagi-Harada syndrome (ICD-10-CM H20.823), although these were expected to be rare.

Patients were followed from 1 year to 10 years after the index date. Outcomes occurring before the 1-year post-index window were not counted (and patients with any such pre-index ocular inflammation were excluded) to ensure we captured incident cases temporally associated with the exposure.

### Statistical analysis

We used Kaplan-Meier survival analysis to estimate the cumulative incidence of outcomes over time, and the log-rank test to compare event-free survival between cannabis users and controls. Cox proportional hazards regression was employed to estimate hazard ratios (HRs) and 95% confidence intervals (CI) for each outcome, comparing the cannabis cohort to the control cohort. The proportional hazards assumption was evaluated for each model, using a significance threshold of *p* < 0.01; results with proportionality *p* < 0.01 were interpreted with caution, as this indicates potential violation of the assumption. Patients without an outcome event were censored at their last known follow-up. A two-tailed *p* < 0.05 was considered statistically significant. All analyses were conducted using the TriNetX Analytics platform.

## Results

The initial database query identified 1,475,532 patients in the cannabis cohort and 3,713,497 patients in the control cohort. After 1:1 propensity score matching, the final analysis included 1,156,655 patients in each group. The matching process achieved excellent balance across baseline characteristics, with mean age approximately 39 years in both groups, similar gender distribution (45% vs. 44% female), and comparable comorbidity profiles. The baseline characteristics are summarized in Table 1.

**Table 1:** Patient characteristics before and after propensity score matching (PSM) for cannabis users compared to non-users in the global collaborative network. PSM, propensity score-matching

Characteristic Name	Before PSM				After PSM			
	Cannabis (*n* = 1,475,532)	Control (*n* = 3,713,497)	*P*	Std diff.	Cannabis (*n* = 1,156,655)	Control (*n* = 1,156,655)	*P*	Std diff.
Age at Index (mean ± SD)	37.08 ± 14.89	52.56 ± 17.5	**< 0.0001**	**0.95**	39.22 ± 15.25	39.01 ± 15.66	< 0.0001	0.01
Female (%)	593,754 (40.56)	1,954,806 (56.09)	**< 0.0001**	**0.31**	516,652 (44.67)	508,230 (43.94)	< 0.0001	0.01
White (%)	814,785 (55.66)	2,166,035 (62.16)	**< 0.0001**	**0.13**	679,124 (58.71)	694,689 (60.06)	< 0.0001	0.03
Black or African American (%)	402,069 (27.46)	488,620 (14.02)	**< 0.0001**	**0.34**	259,018 (22.39)	243,580 (21.06)	< 0.0001	0.03
Hispanic or Latino (%)	123,906 (8.46)	311,878 (8.95)	< 0.0001	0.02	104,728 (9.05)	101,717 (8.79)	< 0.0001	0.01
Not Hispanic or Latino (%)	938,386 (64.1)	2,274,946 (65.28)	< 0.0001	0.02	737,587 (63.77)	745,009 (64.41)	< 0.0001	0.01
Asian (%)	19,823 (1.35)	169,544 (4.86)	**< 0.0001**	**0.20**	19,332 (1.67)	18,657 (1.61)	0.0005	0.00
Hypertensive diseases (%)	217,956 (14.89)	882,730 (25.33)	**< 0.0001**	**0.26**	182,178 (15.75)	191,561 (16.56)	< 0.0001	0.02
Nicotine dependence (%)	293,973 (20.08)	181,432 (5.21)	**< 0.0001**	**0.46**	143,952 (12.45)	140,016 (12.1)	< 0.0001	0.01
Diabetes mellitus (%)	90,040 (6.15)	553,237 (15.88)	**< 0.0001**	**0.31**	83,886 (7.25)	89,117 (7.71)	< 0.0001	0.02
Ischemic heart diseases (%)	60,046 (4.1)	204,650 (5.87)	< 0.0001	0.08	49,104 (4.24)	52,596 (4.55)	< 0.0001	0.01
Chronic kidney disease (%)	35,453 (2.42)	132,390 (3.8)	< 0.0001	0.08	29,219 (2.53)	31,956 (2.76)	< 0.0001	0.01

The primary outcome of any inflammatory eye disease occurred in 1,179 cannabis users compared to 1,078 controls. Kaplan-Meier analysis revealed significantly lower inflammation-free survival in the cannabis group, with cumulative proportion of patients without inflammatory eye disease of 99.53% in cannabis users versus 99.75% in controls (log-rank *p* < 0.0001). The hazard ratio for developing any inflammatory eye disease was 1.8 (95% CI 1.65–1.95, *p* < 0.0001).

**Fig. 1:**
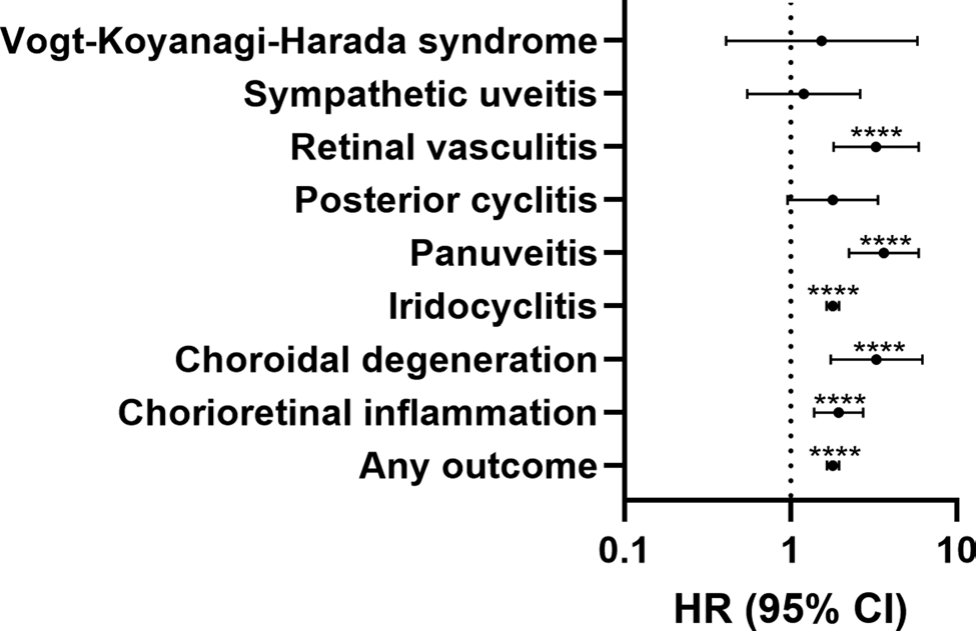
Forest plots summarizing the hazard ratios (HR) with 95% confidence interval (CI) for uveitis and related inflammatory ocular conditions in cannabis users compared to non-users, following propensity score matching. **p < 0.01; ****p < 0.0001

Iridocyclitis was diagnosed in 1,072 cannabis users and 983 controls, with cannabis users showing elevated risk (HR 1.79, 95% CI 1.64–1.96, *p* < 0.0001). Cannabis use demonstrated stronger associations with posterior segment inflammation. Panuveitis occurred in 53 cannabis users versus 24 controls (HR 3.64, 95% CI 2.24–5.91, *p* < 0.0001). Retinal vasculitis was diagnosed in 33 cannabis users versus 17 controls (HR 3.27, 95% CI 1.81–5.89, *p* < 0.0001). Chorioretinal inflammation and choroidal degeneration showed increased risk in cannabis users with HRs of 1.94 (95% CI 1.38–2.73, *p* = 0.0001) and 3.29 (95% CI 1.74–6.23), respectively. Sympathetic ophthalmia, posterior cyclitis and Vogt-Koyanagi-Harada syndrome showed no significant associations with cannabis use. The proportionality assumption was not violated in any of the predefined outcomes. Figure 1; Table 2 provide a detailed presentation of the outcomes between the groups.

**Table 2:** Summary of hazard ratios (HRs) with 95% confidence interval (95% CI), log-rank tests p-values and proportionality tests p-values for uveitis and related inflammatory ocular conditions in cannabis users compared to non-users, following propensity score matching

Outcome	Patients in cohort		Patients with outcome		Survival probability at the end of time window		HR [95% CI]	Log-rank test *p*-value	Proportionality test *p*-value
	Cannabis	Control	Cannabis	Control	Cannabis	Control			
**Iridocyclitis**	1,156,278	1,156,109	1072	983	0.9957	0.9977	1.79 [1.64, 1.96]	< 0.0001	0.0206
Vogt-Koyanagi-Harada syndrome	1,156,652	1,156,652	10	10	1.0000	1.0000	1.54 [0.41, 5.8]	0.522	0.804
**Chorioretinal inflammation**	**1,156,620**	**1,156,594**	**72**	**63**	**0.9997**	**0.9998**	**1.94 [1.38**,** 2.73]**	**0.0001**	**0.3482**
Posterior cyclitis	1,156,648	1,156,647	21	19	0.9999	1.0000	1.8 [0.96, 3.36]	0.0636	0.1083
**Choroidal degeneration**	**1,156,647**	**1,156,647**	**30**	**14**	**0.9999**	**1.0000**	**3.29 [1.74**,** 6.23]**	**0.0001**	**0.1412**
**Retinal vasculitis**	**1,156,647**	**1,156,639**	**33**	**17**	**0.9999**	**1.0000**	**3.27 [1.81**,** 5.89]**	**< 0.0001**	**0.8096**
**Panuveitis**	**1,156,643**	**1,156,641**	**53**	**24**	**0.9998**	**0.9999**	**3.64 [2.24**,** 5.91]**	**< 0.0001**	**0.3144**
Sympathetic uveitis	1,156,633	1,156,589	11	15	0.9999	1.0000	1.2 [0.55, 2.62]	0.6522	0.0698
**Any outcome**	**1,156,234**	**1,156,020**	**1179**	**1078**	**0.9953**	**0.9975**	**1.8 [1.65**,** 1.95]**	**< 0.0001**	**0.012**

## Discussion

This large-scale real-world study provides compelling evidence of an association between cannabis use and ocular inflammatory disease. Cannabis users had a higher hazard of developing any uveitis or inflammatory eye condition compared to non-users, with markedly elevated risks for posterior segment conditions including panuveitis (HR 3.64), choroidal degeneration (HR 3.29) and retinal vasculitis (HR 3.27). These findings suggest cannabis use may be an important and previously under-recognized risk factor for ocular inflammation.

Our findings align with emerging knowledge about cannabis’s pro-inflammatory effects. Laboratory studies demonstrate that cannabis smoke extract can provoke inflammatory responses in human cells, including upregulation of COX-2 and IL-8 [12]. Chronic cannabis use has been associated with systemic inflammation in humans, including increased brain translocator protein (TSPO) levels, a marker of microglial activation consistent with neuroinflammation [13]. 

Cannabis may contribute to ocular inflammation through several pathophysiological mechanisms involving the endocannabinoid system, oxidative stress, and vascular effects. The endocannabinoid system is widely distributed in ocular tissues, including the retina and conjunctiva, where it regulates immune homeostasis and inflammatory responses. Exogenous cannabinoids such as THC and CBD can disrupt this balance, potentially altering immune cell activity and inflammatory mediator production in the eye [10, 11, 14–16]. 

Combustion products from smoked cannabis generate reactive oxygen species and oxidative by-products that induce oxidative stress and promote inflammatory cascades in ocular tissues [17, 18]. The American Heart Association notes that cannabis use, particularly via inhalation, is associated with increased oxidative stress and endothelial dysfunction, which may compromise the blood-retinal barrier and increase vascular permeability, thereby facilitating ocular inflammation [17]. 

Current clinical evidence does not support a strong or consistent association between cannabis use and immune-related eye disorders such as uveitis or thyroid orbitopathy. Recent narrative reviews highlight that while cannabis and cannabinoids have a range of ocular effects-including neuroretinal dysfunction, dry eyes, and transient intraocular pressure reduction-there is insufficient evidence to establish cannabis as a causative factor for immune-mediated ocular diseases, with the literature primarily consisting of case reports and small observational studies that do not provide robust epidemiological evidence [14, 19]. Similarly, regarding the relationship between thyroid disease and uveitis, population-based studies have found only weak to moderate associations, suggesting that shared autoimmune mechanisms may exist but with limited clinical significance. A large case-control study found that patients with thyroid disease had slightly increased odds of uveitis, but the association was not strong enough to warrant routine thyroid screening in uveitis patients [20] while another case-control study found no significant difference in thyroid dysfunction prevalence between patients with non-infectious uveitis and controls, further supporting the lack of a clinically meaningful association [21].

The clinical implications might be significant. Ophthalmologists should consider cannabis use as a potential risk factor when evaluating patients with uveitis, particularly in the absence of other clear etiology. Cannabis users with uveitis may warrant closer monitoring given the elevated risk of sight-threatening complications. From a public health perspective, widespread cannabis use could contribute to increased ocular morbidity on a population level.

Several limitations must be acknowledged. Our analysis relies on ICD-10 diagnostic codes rather than detailed clinical examinations, creating potential for misclassification. As an observational study, we can demonstrate association but not definitive causation, and residual confounding remains possible. We lacked data on cannabis use specifics including quantity, frequency, route of consumption, and potency, which could significantly influence inflammatory impact. Surveillance bias differences between groups might affect diagnosis likelihood.

Future research should focus on prospective studies with regular ophthalmic examinations, dose-response relationships, and comparison of different cannabis forms and cannabinoid ratios. Investigating whether inflammatory risk is reversible upon cessation and how cannabis interacts with standard uveitis treatments would have direct clinical implications. Enhanced patient education about potential ocular risks and incorporation of cannabis use history into routine medical care will be important as legalization continues to expand globally.

## Data Availability

The dataset supporting the conclusions of this article were obtained from the TriNetX Global Collaborative Network, a federated health research platform comprising anonymized electronic medical records from multiple healthcare organizations worldwide. Access to the data is subject to licensing restrictions and cannot be shared publicly. Researchers interested in accessing the data used in this study may apply for access directly through TriNetX (https://www.trinetx.com/), subject to institutional approval and data use agreements.

## Abbreviations

HR: Hazard ratio
CI: Confidence interval
HLA: Human leukocyte antigen
HIPAA: Health Insurance Portability and Accountability Act
ICD-10-CM: International Classification of Diseases, 10th Revision, Clinical Modification
CPT: Current Procedural Terminology
PSM: Propensity score matching
SMD: Standardized mean differences
TSPO: Brain translocator protein
COX2: Cyclooxygenase-2
IL8: Interleukin-8
THC: Tetrahydrocannabinol
CBD: Cannabidiol
ISMS: Information Security Management System
IRB: Institutional Review Board
